# Concurrent Validity and Reliability of Manual Versus Specific Device Transcostal Measurements for Breathing Diaphragm Thickness by Ultrasonography in Lumbopelvic Pain Athletes

**DOI:** 10.3390/s21134329

**Published:** 2021-06-24

**Authors:** Daniel Marugán-Rubio, Jose L. Chicharro, Ricardo Becerro-de-Bengoa-Vallejo, Marta Elena Losa-Iglesias, David Rodríguez-Sanz, Davinia Vicente-Campos, Gabriel J. Dávila-Sánchez, César Calvo-Lobo

**Affiliations:** 1 Faculty of Nursing, Physiotherapy and Podiatry, Universidad Complutense de Madrid, 28040 Madrid, Spain; daniel.marugan@lasallecampus.es (D.M.-R.); ribebeva@ucm.es (R.B.-d.-B.-V.); davidrodriguezsanz@ucm.es (D.R.-S.); cescalvo@ucm.es (C.C.-L.); 2 Grupo FEBIO, Universidad Complutense de Madrid, 28040 Madrid, Spain; jlopezch@ucm.es; 3 Faculty of Health Sciences, Universidad Rey Juan Carlos, 28922 Madrid, Spain; marta.losa@urjc.es; 4 Faculty of Health Sciences, Universidad Francisco de Vitoria, Pozuelo de Alarcón, 28223 Madrid, Spain; 5 Centro Superior de Estudios Universitarios La Salle, 28023 Madrid, Spain; gabriel.davila@lasallecampus.es

**Keywords:** low back pain, repeatability, respiration, ultrasonography, validity

## Abstract

The use of rehabilitative ultrasound imaging (RUSI) to evaluate diaphragm thickness during breathing in athletes who suffer from non-specific lumbopelvic pain presents some measurement errors. The purpose of this study was to evaluate intra- and inter-sessions, intra- and inter-rater reliabilities, and concurrent validity of diaphragm thickness measurements during breathing using transcostal RUSI with a novel thoracic orthotic device that was used to fix the US probe versus those measurements obtained using manual fixation. A total of 37 athletes with non-specific lumbopelvic pain were recruited. Intra- (same examiner) and inter-rater (two examiners) and intra- (same day) and inter-session (alternate days) reliabilities were analyzed. All measurements were obtained after manual probe fixation and after positioning the thoracic orthotic device to fix the US probe in order to correctly correlate both measurement methods. Both left and right hemi-diaphragm thickness measurements were performed by transcostal RUSI at maximum inspiration, expiration, and the difference between the two parameters during relaxed breathing. Intra-class correlation coefficients (ICC), standard errors of measurement (SEM), minimum detectable changes (MCD), systematic errors, and correlations (*r*) were assessed. Orthotic device probe fixation showed excellent reliability (ICC = 0.852–0.996, SEM = 0.0002–0.054, and MDC = 0.002–0.072), and most measurements did not show significant systematic errors (*p* > 0.05). Despite manual probe fixation with a reliability ranging from good to excellent (ICC = 0.714–0.997, SEM = 0.003–0.023, and MDC = 0.008–0.064 cm), several significant systematic measurement errors (*p* < 0.05) were found. Most significant correlations between both orthotic device and manual probe fixation methods were moderate (*r* = 0.486–0.718; *p* < 0.05). Bland–Altman plots indicated adequate agreement between both measurement methods according to the agreement limits. The proposed novel thoracic orthotic device may allow ultrasound probe fixation to provide valid and reliable transcostal RUSI measurements of diaphragmatic thickness during relaxed breathing thus reducing some measurement errors and avoiding systematic measurement errors. It may be advisable to measure diaphragm thickness and facilitate visual biofeedback with respect to diaphragm re-education during normal breathing in athletes with non-specific lumbopelvic pain.

## 1. Introduction

Lumbopelvic pain is considered one of the most common conditions in athletes and has been linked to greater disability and psychological alterations in addition to a poorer quality of life [1,2,3,4,5]. Indeed, lumbopelvic pain suffered by athletes reached point, year, and life prevalence ratios which varied from 10% to 67%, from 17% to 94%, and from 33% to 84%, respectively [6]. In the United States, lumbopelvic pain produces an economic burden of up to 96 million dollars annually [7]. In Europe, the direct economic impact associated with this musculoskeletal condition overcame more than 7000.00 Euros per person [8]. According to gender differences, men and women who suffer from lumbopelvic pain may present different movement patterns in the lumbopelvic region with an earlier activation pattern in men [9]. Athletes who suffer from non-specific lumbopelvic pain present an altered trunk stabilization function secondary to the loss anticipatory activation capacity of the core deep muscles, a process that may impair correct trunk movements, especially in athletes [10].

The stabilizer muscles that comprise the core include transversus abdominis, internal oblique, multifidus, pelvic floor muscles and diaphragm, supporting the motor control and stability for the trunk of athletes. Several tools, such as electromyography, magnetic resonance imaging (MRI), and ultrasound (US) were valid and reliable for assessing static and dynamic functioning under different conditions for which the RUSI technique is considered as a non-invasive, valid, and reliable approach at rest and during activity of the trunk deep stabilizer muscles [11,12]. The benefits of US in musculoskeletal systems have been described for evaluating different soft tissues, such as nerves, joints, tendons, and muscles [13,14].

According to previous studies using US assessments, the RUSI technique was applied to determine static and dynamic functioning of the core deep muscles from athletes, including abdominal wall muscles [15,16,17], multifidus, and low back muscles [18,19,20,21] in addition to pelvic floor muscles [22]. Furthermore, these core muscles were included in rehabilitation and prevention of sports injuries by RUSI visual biofeedback in athletes with lumbopelvic pain [18,19]. Nevertheless, a lack of scientific evidence about morphological and functional changes in the diaphragm muscle during breathing in athletes who suffered from lumbopelvic pain has been found. Indeed, B-mode ultrasonography has shown to be valid and reliable for the transcostal evaluation of the diaphragm morphology and breathing activity [23]. In accordance with these statements, prior MRI studies showed a thinner diaphragm with a reduced excursion during breathing, suggesting an altered muscle motor control in patients with lumbopelvic pain [24]. Patients who suffered from this condition presented greater fatigue [25], decreased excursion, and a higher position of the diaphragmatic dome [26]. However, the RUSI technique was shown to be more portable and cheaper than MRI, justifying its increased use in the physical therapy and rehabilitation fields [11,12].

Indeed, diaphragm training showed positive effects on the lumbar stabilizer muscles of patients who suffered from lumbopelvic pain [27]. Respiratory patterns seemed to play a key role in the athletes’ performances [28]. Furthermore, pelvic floor and diaphragm muscles were shown as synergistic with transversus abdominis muscle and were responsible for maintenance and an increase in intra-abdominal pressure for different postural activities [29]. Despite many studies about the diaphragm, the specific mechanisms that justify the stabilizer role of the diaphragm remain unclear [26]. In addition, alterations in the stabilizing function of the diaphragm seem to be a possible mechanism linked to lumbopelvic pain suffered by athletes, but this finding needs to be further studied [30].

Thus, our research group developed a novel case-control study using the RUSI technique showing a bilateral reduction of the diaphragm thickness upon inspiration and a decreased thickness change during breathing without differences in the diaphragmatic excursion in athletes with lumbopelvic pain compared to healthy athletes [30]. The manual procedure used in this study to fix the US probe during diaphragm thickness evaluations showed excellent reliability. Nevertheless, the standard error of measurements (SEMs) and minimum detectable change (MDCs) values in conjunction with the small thickness changes of the diaphragm during breathing assessed by transcostal RUSI presented some difficulties when interpreting the resulting differences that in some cases exceeded measurement errors even when expert evaluators performed the measurements. Some custom-made ultrasound probe holders were designed to measure abdominal and hip muscle thickness changes during functional activities [31,32] although prior studies have not yet applied thoracic orthoses and ultrasound probe holders in the thoracic region to assess diaphragmatic thickness. These issues justified the development of a specific thoracic orthotic device in order to reduce measurement errors of the diaphragm thickness assessed by transcostal RUSI using a probe manual fixation. Thus, we hypothesized that the novel thoracic orthotic device could allow fixation of the US probe to facilitate a valid and reliable transcostal evaluation of the diaphragmatic thickness during breathing in order to reduce measurement errors introduced by manual fixation of the probe. Finally, our purpose was to determine the intra- and inter-sessions and intra- and inter-rater reliabilities as measured by transcostal RUSI in addition to concurrent validity of diaphragm thickness measurements obtained during breathing from this thoracic orthotic device versus manual fixation used to fix the US probe in athletes with non-specific lumbopelvic pain.

## 2. Methods

### 2.1. Study Design and Patent Registry

The present reliability and concurrent validity study was carried out from July 2020 to April 2021 following the Guidelines for Reporting Diagnostic Accuracy (STARD) criteria [33]. The Helsinki Declaration and human experimentation ethical requirements were respected [34]. This study was approved by the clinical intervention ethics committee of the Hospital Clínico San Carlos, Madrid (Spain) with internal code number 19/421-E_BS on 9 October 2019. All participants signed an informed consent form before participating in the study.

Previously, a patent was registered as a utility model for a holding device for a US probe in the Spanish Patent and Trademark Office (Application number: U202000080; Publication number: ES1245754; Issue Date: 24 August 2020). This holding device was used to fix the US probe within a thoracic orthotic device in order to reduce measurement errors of the diaphragm thickness assessed by transcostal RUSI using manual fixation of the US probe [30]. This work was supported by the Madrid Government (Comunidad de Madrid, Spain) under the Multiannual Agreement with Complutense University (Reference project number PR65/19-22348) in the line Program to Stimulate Research for Young Doctors in the context of the V PRICIT (Regional Program of Research and Technological Innovation).

### 2.2. Sample Size Calculation

An a priori sample size calculation was carried out by means of bivariate correlation statistical tests in order to obtain concurrent validity using the G*Power 3.1.9.2 software (G*Power©, University of Dusseldorf, Dusseldorf, Germany) using a correlation coefficient of 0.4 to determine a moderate correlation [35] between RUSI transcostal measurements of the diaphragm thickness during breathing [30] obtained by using the thoracic orthotic device to fix the US probe compared to the measurements obtained after manual fixation of the probe, considering one-tailed hypothesis, α error of 0.05, and a power of 0.80 (1-β error probability). According to these parameters, a sample size calculation of 37 subjects was required to achieve an actual power of 0.806.

In addition, an a posteriori sample size calculation was performed by the difference between two dependent means using statistical *t*-tests in order to justify systematic errors of measurements between matched pairs using the same G*Power 3.1.9.2 software (G*Power©, University of Dusseldorf, Germany). A medium effect size of 0.5 was applied [36], also considering a one-tailed hypothesis, α error of 0.05, and a power of 0.80 (1-β error probability). Regarding these parameters, a sample size calculation of 27 subjects was necessary to obtain an actual power of 0.811.

### 2.3. Patients

A total of 37 athletes who suffered from non-specific lumbopelvic pain were recruited via consecutive sampling. Inclusion criteria consisted of athletes with bilateral non-specific lumbopelvic pain for at least six weeks with pain distribution from the iliac crest to the popliteal fossa, showing positive bilateral active straight leg test, amateur or semiprofessional sport activity level (training at least two hours of training and one day per week in addition to playing one match or competition per week), moderate (II-level) or vigorous (III-level) physical activity within a metabolic equivalent index greater than 600 METs/min/week, according to the International Physical Activity Questionnaire (IPAQ), and age between 18 and 65 years [30]. Exclusion criteria included lumbopelvic congenital alterations, rheumatic or neuromuscular conditions, body mass index (BMI) > 31 kg/m^2^, previous diagnoses regarding respiratory or neurological conditions, surgeries, lower extremity pathologies (such as sprains, fractures, or chronic ankle instability), skin alterations, and inability to follow the instructions to complete the correct study course. In addition, rest and physical reductions of fitness or daily exercise for more than four weeks in addition to the presence of hyperventilation syndrome assessed by the Nijmegen’s test with a score ≥24 points were also considered as exclusion criteria [16,30].

### 2.4. Procedure

**Figure 1:**
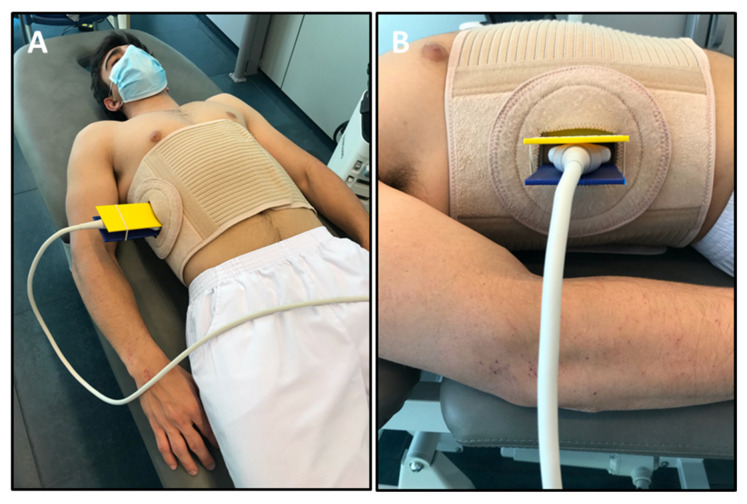
Thoracic orthotic device to measure diaphragm thickness. (**A**) Thoracic orthosis with the holding device to fix the ultrasound (US) probe. (**B**) Support and fixation adapter to place the linear US probe perpendicular to the last intercostal space along the mid-axillary line.

Initially, the holding device was positioned to fix the US probe within a thoracic orthotic device in order to reduce measurement errors of the diaphragm thickness as assessed by transcostal RUSI using manual probe fixation [30]. This device allows total thoracic mobility and includes a space to incorporate US gel for a complete visualization of the last intercostal space. In addition, a support and fixation adapter (patent utility model registry: U202000080) was included in order to place the linear ultrasound probe perpendicular to the last intercostal space along the mid-axillary line (Figure 1). On the one hand, the procedure of fixing and positioning the probe was carried out by manual probe fixation with the participant placed at the supine decubitus in order to measure bilaterally diaphragm thickness positioning the probe in the last intercostal space according to Harper et al. [23]. On the other hand, the procedure of fixing and positioning the probe was performed with a thoracic orthotic device in the same described position with the participant placed at the supine decubitus position. The orthotic device was placed at the last intercostal space of the right and left hemi-diaphragm coinciding with the bivalve adapter in which the US probe holder was inserted in order to fix the probe as shown in Figure 1A,B. Intra- (same examiner) and inter-rater (two examiners) reliabilities and intra- (same day separated by one hour) and inter-session (alternate days separated by 48 h) reliabilities were analyzed [37]. All measurements were performed via manual probe fixation and with the thoracic orthotic device used to fix the ultrasound probe in order to correlate both measurement methods for concurrent validity analyses. Both examiners had >4 years of experience working with the RUSI technique and evaluated bilateral diaphragm thickness during relaxed breathing by a randomized assessment order regarding examiners and hemi-diaphragm and measurement method. RUSI images were coded, saved, and later analyzed using a blinding procedure performed by a blinded examiner who measured diaphragm US thickness of the coded images using the ImageJ software [30].

### 2.5. Descriptive Data

Descriptive data, such as gender (male or female), age (years), height (cm), weight (kg), BMI (kg/cm^2^ according to Quetelet´s index) [38], sport category (fitness, running, triathlon, ballet, or rugby), dominant side, throwing hand-side dominance and jumping foot dominance (right or left), and smoking habits (yes or no) were detailed [15,21,30]. According to the International Physical Activity Questionnaire (IPAQ), which presented acceptable psychometric properties, the metabolic equivalent index per minute per week (METs/min/week) was measured to quantify physical activity and categorized as moderate (<1500 METs/min/week) or vigorous (≥1500 METs/min/week) [39]. Respiratory distress was measured using the Nijmegen’s questionnaire, which has been previously used in RUSI evaluations in patients with non-specific lumbopelvic pain due to its possible relationship with diaphragm-related activity [21,30].

### 2.6. Clinical Data

Pain intensity was measured with the visual analogue scale (VAS) in a horizontal line (mm) from 0 (no pain) to 100 (worst pain), which has shown an adequate reliability (intraclass correlation coefficient [ICC] = 0.65–0.88) and concurrent validity (*r* = 0.74) [4,40]. The pressure pain threshold (PPT) was bilaterally assessed in the center of the paraspinal muscles at the L3 vertebrae processus spinosus from 0 to 10 kg/cm^2^ with a mechanical algometer (Wagner Instruments, Greenwich, CT) using the mean of three repeated measurements within a 30 to 60 s interval. This process presented adequate measurement properties (CCI = 0.91, variation coefficient = 10.3%, SEM = 0.19 kg/cm^2^, and MDC = 0.54 kg/cm^2^) [20,41]. Disability linked to lumbopelvic pain was evaluated by the Roland–Morris Disability Questionnaire (RMDQ), whose Spanish version was shown to be valid and reliable (ICC = 0.87) [30]. The short-form 12-item (SF-12) health questionnaire was used to measure health-related quality of life (QoL) in score and optimal normalized values by applying physical health and mental health domains and total score, which showed adequate validity and reliability psychometric properties (α Cronbach = 0.78–0.85) [42].

### 2.7. Ultrasound Measurements

**Figure 2:**
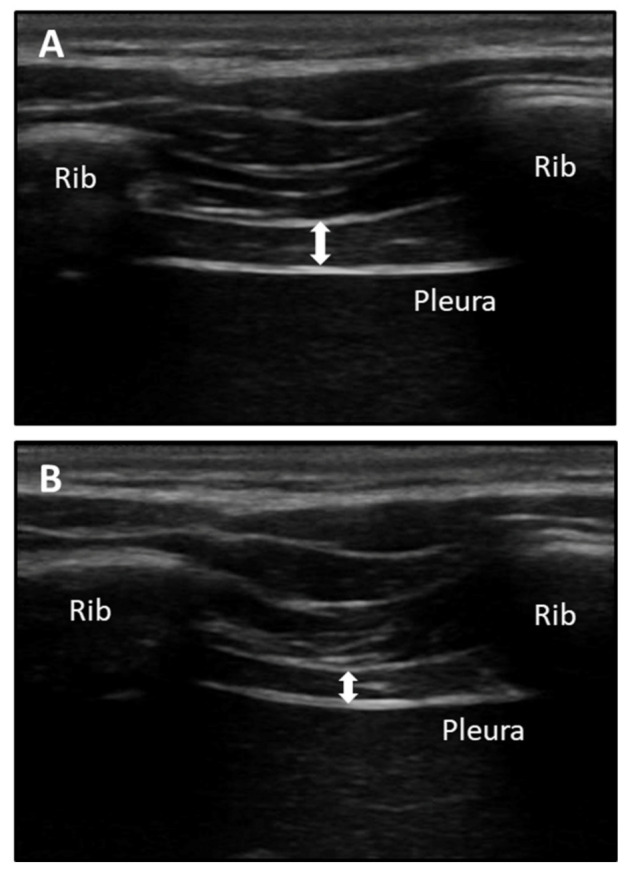
B-mode diaphragm thickness ultrasound imaging visualizing the last intercostal space following the mid-axillary line from the inferior edge of the 11th rib to the superior edge of the de la 12th rib of the thorax. (**A**) Diaphragm thickness (white arrow) at maximum inspiration (T^ins^) during relaxed breathing. (**B**) Diaphragm thickness (white arrow) at maximum expiration (T^exp^) during relaxed breathing.

Both left and right hemi-diaphragm thickness measurements (cm) were performed using the transcostal RUSI technique at maximum inspiration (T^ins^), maximum expiration (T^exp^), and the difference between these two parameters (T^ins-exp^) during relaxed breathing. A high-quality US tool (Ecube i7; Alpinion Medical System; Seoul, Korea) was used to measure all US images. A linear probe (L3_12T type; field of view of 38.4 mm; 128 elements) with a frequency range from 8 to 12.0 MHz and a probe footprint of 45 mm were used to measure diaphragm thickness while the patient was in a supine position via B-mode US imaging with a prefixed preset (depth of 3 cm, frequency of 12 MHz, gain of 64 points, dynamic range of 64 points, and one focus located at a depth of 2 cm) [23,30]. Gray-scale images for RUSI measurements were converted in Digital Imaging and Communications in Medicine (DICOM), transferred to a computer, and calibrated in cm, by the 2.0 v ImageJ software (National Institutes of Health; Bethesda, MD, USA) in order to measure the outline of the diaphragm thickness [30]. Indeed, the linear probe was perpendicularly placed with respect to the last intercostal space following the mid-axillary line from the inferior edge of the 11th rib to the superior edge of the 12th rib of the thorax, which permitted adequate visualization of the diaphragm under the intercostal muscle connective tissue during relaxed breathing (Figure 2). A total of three repeated measurements were obtained for diaphragm thickness (left and right thickness at T^ins^, T^esp^, and T^ins-esp^) for a total of three images for each parameter. Thickness measurements were performed by placing electronic calipers inside the hyperechogenic lines of the perimuscular connective tissue, which outlined the diaphragm muscle in the center of the intercostal space. The mean of three repeated measurements was calculated. This procedure has shown excellent intra- (CCI = 0.93–0.96) and inter-rater (ICC = 0.97–0.98) reliabilities [23,30]. Nevertheless, the development of the proposed thoracic orthosis device was intended to reduce measurement errors (EEM = 0.23–0.72 cm) and minimum detectable changes (CMD = 0.06–2.00 cm) as shown in our previously published analyses [30].

### 2.8. Statistical Analyses

In order to carry out all statistical analyses, the 24 v Statistical Package of Social Sciences (SPSS) software (IBM, Armonk, NY, USA, IBM-Corp) was used applying an α error of 0.05 and a *p*-value < 0.05, which was considered statistically significant for a 95% confidence interval (CI). The Kolmogorov–Smirnov test was used to assess normality. Data were described as mean ± standard deviation (SD) completed with upper and lower limits of the 95% CI. Outcome measurement comparisons for sessions and raters were assessed by the Student’s *t*-test for paired samples for parametric data and the Wilcoxon test for non-parametric data. The reliability and concurrent validity between two measurements was determined by the ICC for bidirectional absolute agreement and Pearson (*r* for parametric data) or Spearman (*r*_s_ for non-parametric data) correlation coefficient, respectively. The ICC_(2,1)_ was considered for intra- and inter-examiner reliability in addition to intra- and inter-session reliability according to a previously published study about US imaging evaluations [43]. The values for ICC_(2,1)_ were interpreted as poor (ICC_(2,1)_ < 0.40), weak (ICC_(2,1)_ = 0.40–0.59), good (ICC _(2,1)_ = 0.60–0.74), or excellent (ICC_(2,1)_ = 0.75–1.00) [30]. Furthermore, Pearson or Spearman coefficient correlation coefficients were interpreted as week (*r* or *r*_s_ = 0.00–0.40), moderate (*r* or *r*_s_ = 0.41 – 0.69), and strong (*r* or *r*_s_ = 0.70–1.00) [35]. SEM values were calculated according to SEM = SD × $\sqrt{\left(1-ICC\right)}$ [44]. In addition, the MDC was calculated according to MDC = $\sqrt{2}\times 1.96\times SEM$ for a 95% CI. Both SEM and MDC were analyzed according to Bland and Altman [30]. The limits of agreement (LoA) between both manual and specific orthotic device measurement methods were calculated by the formula LoA = difference mean ± 1.96×SD for a 95% CI according to Bland and Altman [30,45]. Finally, Bland–Altman plots were performed in order to determine the agreement between both manual and specific orthotic device measurement methods showing the visual distribution of systematic measurement error of the mean of the difference between each pair of measurements on the *y*-axis versus the mean for each pair of measurements on the *x*-axis [45].

## 3. Results

### 3.1. Descriptive Data

The sample consisted of 25 (67.6%) male and 12 (32.4%) female athletes with non-specific lumbopelvic pain, who presented a mean ±SD (95% CI) of 31.64 ± 5.56 (29.79−33.50) years, 173.45 ± 6.93 (171.14–175.77) cm, 70.18 ± 10.55 (66.58–73.62) kg, and 23.14 ± 2.37 (22.35–23.93) kg/cm^2^. The main sport undertaken by these athletes was fitness (n = 32; 86.5%), running (n = 2; 5.4%), triathlons (n = 1; 2.7%), ballet (n = 1; 2.7%), and rugby (n = 1; 2.7%). Most athletes presented the right dominant side (n = 32; 86.5%), right throwing hand-side dominance (n = 34; 91.9%), and right jumping foot dominance (n = 31; 83.8%) and were non-smokers (n = 31; 83.8%). According to the IPAQ, the metabolic equivalent index per minute per week mean ±SD (95% CI) was 2665.94 ± 1729.30 (2089.36–3242.52) METs/min/week, and 28 (75.7%) athletes carried out vigorous physical activity, while only 6 (16.2%) performed moderate physical activity. Athletes who did not report respiratory distress according to Nijmegen’s questionnaire had a mean ±SD (95% CI) of 11.21 ± 5.82 (9.27–13.15).

### 3.2. Clinical Data

Athletes suffering from non-specific lumbopelvic pain presented a mean ±SD (95% CI) of VAS pain intensity of 5.27 ± 5.72 (5.30–6.15) points, PPT of 4.65 ± 1.26 (4.23–5.08) kg/cm^2^, and 4.72 ± 1.47 (4.23–5.22) kg/cm^2^ for the right and left paraspinal muscles, respectively. The RMDQ disability score was 3.40 ± 3.51 (2.23–4.57) points, and SF-12 score and normalized optimal values for physical health were 17.02 ± 2.00 (16.35–17.69) and 78.83 ± 14.44 (74.02 ± 83.65) points, respectively, mental health 20.83 ± 3.97 (19.51–22.16) and 70.32 ± 18.68 (64.09–76.55), and total score and an optimal value of 37.81 ± 5.15 (36.09–39.52) and 73.54 ± 14.96 (68.55–78.52), respectively.

### 3.3. Intra-Rater and Intra-Session Reliability and Concurrent Validity for RUSI Diaphragm Thickness

The orthotic device probe fixation caused an improvement in intra-rater and intra-session reliability for RUSI diaphragm thickness with an excellent reliability (ICC_(1,2)_ = 0.935–0.996) and measurement errors (SEM = 0.0002–0.012 cm; MDC = 0.005–0.035 cm) and did not show any statistically significant systematic error of measurement (*p* > 0.05). Nevertheless, the manual probe fixation presented intra-rater and intra-session reliabilities ranging from good to excellent (ICC_(1,2)_ = 0.714–0.994), a larger range of measurement errors (SEM = 0.003–0.021 cm; MDC = 0.008–0.059 cm), and statistically significant systematic errors of measurement (*p* < 0.05) for left diaphragm thickness at T^exp^ and T^ins-exp^ (Table 1).

Regarding intra-rater and intra-session measurements, all correlations between both orthotic device and manual probe fixation methods were moderate for the right (*r* = 0.489 –0.692; *p* < 0.01) and left (*r* = 0.564–0.654; *p* < 0.001) diaphragm thickness measurements.

### 3.4. Intra-Rater and Inter-Session Reliabilities and Concurrent Validity for RUSI Diaphragm Thickness

The orthosis device probe fixation showed excellent intra-rater and inter-session reliabilities for RUSI diaphragm thickness (ICC_(1,2)_ = 0.98–0.993), SEM = 0.003–0.012 cm, MDC = 0.010–0.021 cm) and did not show any statistically significant systematic error of measurement (*p* > 0.05). Similarly, the manual probe fixation presented excellent intra-rater and inter-session reliabilities (ICC_(1,2)_ = 0.982–0.997), SEM = 0.003–0.006 cm, MDC = 0.008–0.018 cm) and did not show any statistically significant systematic error of measurement (*p* > 0.05) as shown in Table 2.

Considering intra-rater and inter-session measurements, correlations between both orthotic device and manual probe fixation methods were moderate for the right (*r* = 0.501–0.718; *p* < 0.01) and left (*r* = 0.586–0.628; *p* < 0.001) diaphragm thickness measurements but not significant and weak for left diaphragm at T^ins-exp^ (*r* = 0.257; *p* = 0.125).

### 3.5. Inter-Rater and Intra-Session Reliabilities and Concurrent Validity for RUSI Diaphragm Thickness

The orthotic device probe fixation showed an excellent inter-rater and intra-session reliability for RUSI diaphragm thickness (ICC_(1,2)_ = 0.875–0.982), SEM = 0.001–0.017 cm, MDC = 0.002 0.047 cm) and only showed a statistically significant systematic error of measurement for the left diaphragm thickness at T^exp^ (*p* < 0.05). Similarly, the manual probe fixation presented excellent inter-rater and intra-session reliabilities (ICC_(1,2)_ = 0.872–0.983), SEM = 0.008–0.016 cm, MDC = 0.024–0.044 cm) and showed more statistically significant systematic errors of measurement (*p* < 0.05) for the right and left diaphragm thickness at T^exp^ and T^ins-exp^ (Table 3).

Regarding inter-rater and intra-session measurements, correlations between both orthotic device and manual probe fixation methods were moderate for the right (*r* = 0.486–0.638; *p* < 0.01) and left (*r* = 0.637–0.658; *p* < 0.001) diaphragm thickness measurements, but not significant and weak for left diaphragm at T^ins-exp^ (*r* = 0.237; *p* = 0.158).

### 3.6. Inter-Rater and Inter-Session Reliabilities and Concurrent Validity for RUSI Diaphragm Thickness

The orthotic device probe fixation showed excellent inter-rater and inter-session reliabilities for RUSI diaphragm thickness (ICC_(1,2)_ = 0.852–0.927), SEM = 0.013–0.054 cm, and MDC = 0.027–0.072 cm) and did not show any statistically significant systematic error of measurement (*p* > 0.05). Despite excellent inter-rater and inter-session reliabilities of the manual probe fixation method (ICC_(1,2)_ = 0.784–0.965, SEM = 0.013–0.023 cm, and MDC = 0.036–0.064 cm), statistically significant systematic errors of measurement (*p* < 0.05) for the right diaphragm thickness at T^ins^, T^exp^ and T^ins-exp^, and left diaphragm thickness at T^exp^ were found (Table 4).

According to inter-rater and inter-session measurements, correlations between both orthotic device and manual probe fixation methods were moderate for the right (*r* = 0.553–0.652; *p* < 0.001) and left (*r* = 0.637–0.658; *p* < 0.001) diaphragm thickness measurements but were not significant and weak for left diaphragm at T^ins-exp^ (*r* = 0.169; *p* = 0.318).

**Figure 3:**
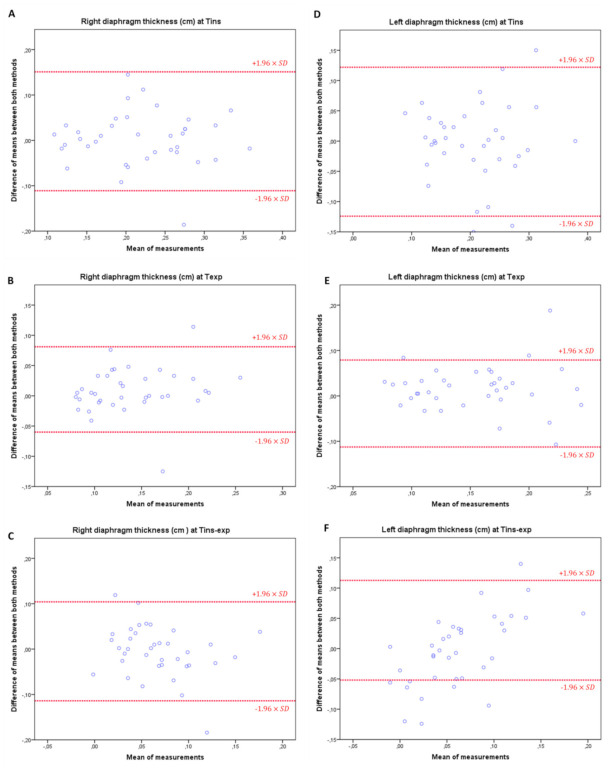
Bland-Altman plots agreement between both manual and specific orthosis device measurement methods for the right and left diaphragm thickness at T^ins^, T^exp^ and T^ins-exp^, completed with the upper and lower limits of agreement (LoA). (**A**) Right diaphragm thickness at maximum inspiration (T^ins^) during relaxed breathing. (**B**) Right diaphragm thickness at maximum expiration (T^exp^) during relaxed breathing. (**C**) Right diaphragm thickness difference (T^insp-exp^) during relaxed breathing. (**D**) Left diaphragm thickness at maximum inspiration (T^ins^) during relaxed breathing. (**E**) Left diaphragm thickness at maximum expiration (T^exp^) during relaxed breathing. (**F**) Left diaphragm thickness difference (T^insp-exp^) during relaxed breathing.

Finally, Bland–Altman plots demonstrated an adequate agreement between both manual and specific orthotic device measurement methods for the right and left diaphragm thickness at T^ins^, T^exp^ and T^ins-exp^ due to visual distribution of the difference mean between each pair of measurements on the Y axis versus the mean for each pair of measurements on the X axis, which did not show systematic measurement errors (Figure 3). Most measurements were between upper and lower limits of agreement (LoA).

## 4. Discussion

The proposed novel thoracic orthotic device may allow fixation of a US probe and provide valid and reliable transcostal RUSI measurements of the diaphragmatic thickness during relaxed breathing thus reducing some measurement errors and avoiding systematic errors of measurement with respect to measurements when using manual fixation of the probe. Thus, intra- and inter-session and intra- and inter-rater reliabilities in addition to concurrent validity of diaphragm thickness measurements during breathing by transcostal RUSI carried out by this thoracic orthosis device that was used to fix the US probe seemed to be adequate and may be more advisable with respect to the measurements performed by manual fixation of the probe in athletes with non-specific lumbopelvic pain.

This device could reduce measurement errors according to the problems reported by our research group during evaluation of diaphragm thickness during normal breathing as assessed with the manual fixation of the probe by the RUSI technique in athletes with lumbopelvic pain [30]. These issues justified the development of this utility model patent for this thoracic orthosis device by reducing some measurement errors and avoiding systematic differences of the measurements of the diaphragm thickness assessed by transcostal RUSI using a probe manual fixation.

Overall, reliability for the RUSI diaphragm thickness as measured with the orthosis device probe fixation showed similar adequate reliability and led to slightly reduced measurements errors, especially during inspiration at both right and left hemi-diaphragms compared to the manual probe fixation. Nevertheless, some measurement errors, including expiration measurements with the orthotic device, were similar or greater than the manual probe fixation measurement. This issue may have been due to consideration of the diaphragm muscle as the key inspiratory muscle during postural activity [24] since different artifacts and paradoxical respiration patterns were specially reported at rest during expiration in athletes with lumbopelvic pain [30].

In addition, several systematic errors of measurement were reported within the manual probe fixation for both hemi-diaphragms, which occurred more often during expiration evaluations, while only one systematic error of measurement was presented within the orthotic device at the left hemi-diaphragm during expiration. These findings were in line with our previous study in which some problems in the interpretation of the left diaphragm thickness differences in athletes with lumbopelvic pain, showing more common paradoxical respiration patterns in the left hemi-diaphragm, were detailed [30]. A plausible reason for these results may be that the left hemi-diaphragm presented a higher diaphragm muscle thickness variability [23] in addition to lower excursion and higher position of its dome in patients with lumbopelvic pain [26]. Furthermore, this variability could explain controversial findings obtained in different intra- and inter-session and intra- and inter-examiner comparisons of our study concerning reliability and measurement and systematic errors.

In addition, the proposed holding device used to fix the US probe in a thoracic orthotic device may reduce measurement errors of the diaphragm thickness as assessed by transcostal RUSI in conjunction with the facilitation of visual diaphragm re-education during normal breathing thus avoiding the problems related to the use of a probe manual fixation. Previous studies have suggested an altered diaphragm muscle motor control in patients with lumbopelvic pain [24], greater fatigue [25], and decreased excursion and a higher position of the diaphragmatic dome [26], which could be improved by visual diaphragm re-education during normal breathing. Re-education of the breathing patterns may modify these respiratory patterns by improving the range of motion, improving diaphragm muscle contraction, and decreasing accessory muscle activity in patients who suffer from musculoskeletal disorders [46]. Therefore, this device could help to improve postural re-education and motor control, which would improve pain and disability in patients with lumbopelvic pain [47,48].

### 4.1. Future Studies

This novel tool should be studied in other musculoskeletal conditions that are different from lumbopelvic pain, such as neck pain [46] or heart failure [49,50] since inspiratory muscle training has shown great benefits in these patients. In addition, randomized clinical trials assessing the effects of diaphragm re-education based on visual biofeedback using this novel holding device for the probe fixation could improve signs and symptoms shown by athletes who are suffering from non-specific lumbopelvic pain [30,47,48]. In addition to this device to measure diaphragm thickness and the US holders designed to assess abdominal and gluteus muscles [31,32], new US probe holders should be designed to measure trunk deep stabilizer muscles due to their high clinical value in athletes with lumbopelvic pain [21].

### 4.2. Limitations

Some limitations need to be considered for the use of the proposed device. Despite the reduction in diaphragm thickness measurements and systematic errors after using the thoracic orthotic device compared to manual fixation of the probe during normal breathing, special caution should be paid while using the novel holding device to measure left diaphragm thickness. Systematic errors of measurement for inter-rater and intra-session reliabilities at T^exp^ and non-significant correlations for concurrent validity at T^ins-exp^ have been found. In addition, *t*-tests were not applied to compare both methodologies because we prioritized correlation analyses between both manual and thoracic orthotic device measurements in order to uphold concurrent validity according to our previously described sample size calculation by means of bivariate correlation statistical tests. Despite manual probe fixation consideration in the present study as the reference for diaphragm thickness measurements in athletes with lumbopelvic pain [30], future concurrent validity studies should consider MRI as the gold standard [26]. Finally, our sample consisted of patients with an age ranging from 29.79–33.50 years, and the authors encourage researchers to study heterogeneous cohorts in terms of age due to the influence of age on muscle thickness [51].

## 5. Conclusions

The proposed novel thoracic orthotic device may allow fixation of the US probe thus providing valid and reliable transcostal RUSI measurements of the diaphragmatic thickness during relaxed breathing and may cause a reduction in some measurement errors by avoiding systematic errors of measurement with respect to measurements carried out by manual probe fixation. Thus, this holding device for the US probe may be recommended for measuring diaphragm thickness and facilitating visual biofeedback re-education of the diaphragm during normal breathing in athletes with non-specific lumbopelvic pain.

## Figures and Tables

**Table 1:** Intra-rater and intra-session reliability analysis for RUSI diaphragm thickness within both manual and specific orthosis device measurement methods during relaxed breathing.

RUSI Diaphragm Thickness (cm)	Baseline Mean ± SD (95% CI)	After 1 h Mean ± SD (95% CI)	ICC_(1,2)_ (95% CI)	SEM	MDC	*p*-Value
**Manual Probe Fixation**						
T^ins^ right diaphragm	0.21 ± 0.07 (0.19–0.24)	0.22 ± 0.07 (0.19–0.24)	0.989 (0.978–0.994)	0.009	0.024	0.141 ^†^
T^exp^ right diaphragm	0.13 ± 0.04 (0.11–0.15)	0.13 ± 0.04 (0.12–0.15)	0.993 (0.986–0.996)	0.003	0.008	0.141 *
T^ins-exp^ right diaphragm	0.08 ± 0.05 (0.06–0.10)	0.08 ± 0.05 (0.06–0.10)	0.982 (0.966–0.991)	0.006	0.018	0.404 *
T^ins^ left diaphragm	0.20 ± 0.07 (0.18–0.23)	0.21 ± 0.07 (0.18–0.23)	0.994 (0.989–0.997)	0.005	0.015	0.152 *
T^exp^ left diaphragm	0.15 ± 0.05 (0.13–0.17)	0.10 ± 0.03 (0.09–0.11)	0.714 (−0.11–0.92)	0.021	0.059	<0.001 *
T^ins-exp^ left diaphragm	0.05 ± 0.04 (0.04–0.06)	0.10 ± 0.04 (0.09–0.12)	0.982 (0.966–0.991)	0.005	0.014	<0.001 *
**Orthosis Device Probe Fixation**						
T^ins^ right diaphragm	0.23 ± 0.07 (0.20–0.25)	0.23 ± 0.07 (0.20–0.26)	0.991 (0.982–0.995)	0.006	0.018	0.327 *
T^exp^ right diaphragm	0.15 ± 0.05 (0.13–0.17)	0.16 ± 0.05 (0.14–0.18)	0.947 (0.892–0.973)	0.002	0.007	0.067 ^†^
T^ins-exp^ right diaphragm	0.07 ± 0.05 (0.05–0.09)	0.07 ± 0.05 (0.05–0.09)	0.935 (0.874–0.966)	0.012	0.035	0.283 ^†^
T^ins^ left diaphragm	0.22 ± 0.07 (0.19–0.24)	0.22 ± 0.07 (0.19–0.24)	0.993 (0.987–0.997)	0.005	0.015	0.823 *
T^exp^ left diaphragm	0.17 ± 0.05 (0.15–0.19)	0.17 ± 0.05 (0.15–0.19)	0.996 (0.992–0.998)	0.0002	0.0005	0.119 *
T^ins-exp^ left diaphragm	0.04 ± 0.05 (0.03–0.06)	0.04 ± 0.04 (0.03–0.06)	0.978 (0.958–0.989)	0.006	0.018	0.360 *

Abbreviations: CI, confidence interval; ICC, intraclass correlation coefficient; MDC, minimum detectable change; SD, standard deviation; SEM, standard error of measurement; T^ins^, maximum inspiration time; T^exp^, maximum expiration time. *p* < 0.05 was considered as statistically significant for a 95% CI (**in bold**). * Student *t*-test for paired samples was used. ^†^ Wilcoxon test for paired samples was used.

**Table 2:** Intra-rater and inter-session reliability analysis for RUSI diaphragm thickness within both manual and specific orthosis device measurement methods during relaxed breathing.

RUSI Diaphragm Thickness (cm)	Baseline Mean ± SD (95% CI)	After 48 h Mean ± SD (95% CI)	ICC_(1,2)_ (95% CI)	SEM	MDC	*p*-Value
**Manual Probe Fixation**						
T^ins^ right diaphragm	0.21 ± 0.07 (0.19–0.24)	0.22 ± 0.07 (0.19–0.24)	0.992 (0.985–0.996)	0.006	0.018	0.306 ^†^
T^exp^ right diaphragm	0.13 ± 0.04 (0.11–0.15)	0.13 ± 0.04 (0.12–0.15)	0.993 (0.985–0.996)	0.003	0.008	0.050 *
T^ins-exp^ right diaphragm	0.08 ± 0.05 (0.06–0.10)	0.08 ± 0.06 (0.06–0.10)	0.985 (0.971–0.992)	0.006	0.018	0.766 *
T^ins^ left diaphragm	0.20 ± 0.07 (0.18–0.23)	0.20 ± 0.07 (0.18–0.23)	0.997 (0.995–0.999)	0.003	0.010	0.397 *
T^exp^ left diaphragm	0.15 ± 0.05 (0.13–0.17)	0.15 ± 0.05 (0.13–0.17)	0.992 (0.985–0.996)	0.004	0.012	0.838 *
T^ins-exp^ left diaphragm	0.05 ± 0.04 (0.04–0.06)	0.05 ± 0.04 (0.03–0.06)	0.982 (0.965–0.991)	0.005	0.014	0.491 *
**Orthosis Device Probe Fixation**						
T^ins^ right diaphragm	0.23 ± 0.07 (0.20–0.25)	0.23 ± 0.07 (0.20–0.26)	0.993 (0.986–0.996)	0.005	0.016	0.173 *
T^exp^ right diaphragm	0.15 ± 0.05 (0.13–0.17)	0.16 ± 0.05 (0.14–0.18)	0.941 (0.881–0.970)	0.003	0.010	0.265 ^†^
T^ins-exp^ right diaphragm	0.07 ± 0.05 (0.05–0.09)	0.07 ± 0.05 (0.05–0.09)	0.933 (0.870–0.965)	0.012	0.035	0.317 ^†^
T^ins^ left diaphragm	0.22 ± 0.07 (0.19–0.24)	0.22 ± 0.07 (0.19–0.24)	0.990 (0.981–0.995)	0.006	0.018	0.776 *
T^exp^ left diaphragm	0.17 ± 0.05 (0.15–0.19)	0.17 ± 0.05 (0.15–0.18)	0.982 (0.965–0.991)	0.006	0.018	0.587 *
T^ins-exp^ left diaphragm	0.04 ± 0.05 (0.03–0.06)	0.04 ± 0.04 (0.03–0.06)	0.961 (0.925–0.980)	0.007	0.021	0.840 *

Abbreviations: CI, confidence interval; ICC, intraclass correlation coefficient; MDC, minimum detectable change; SD, standard deviation; SEM, standard error of measurement; T^ins^, maximum inspiration time; T^exp^, maximum expiration time. *p* < 0.05 was considered as statistically significant for a 95% CI. * Student *t*-test for paired samples was used. ^†^ Wilcoxon test for paired samples was used.

**Table 3:** Inter-rater and intra-session reliability analysis for RUSI diaphragm thickness within both manual and specific orthosis device measurement methods during relaxed breathing.

RUSI Diaphragm Thickness (cm)	Examiner 1 Mean ± SD (95% CI)	Examiner 2 Mean ± SD (95% CI)	ICC_(2,1)_ (95% CI)	SEM	MDC	*p*-Value
**Manual Probe Fixation**						
T^ins^ right diaphragm	0.21 ± 0.07 (0.19–0.24)	0.21 ± 0.07 (0.19–0.24)	0.983 (0.967–0.991)	0.009	0.025	0.174 ^†^
T^exp^ right diaphragm	0.13 ± 0.04 (0.11–0.15)	0.12 ± 0.04 (0.10–0.13)	0.951 (0.774–0.982)	0.008	0.024	<0.001 *
T^ins-exp^ right diaphragm	0.08 ± 0.05 (0.06–0.10)	0.09 ± 0.06 (0.07–0.11)	0.955 (0.889–0.979)	0.011	0.031	0.004 *
T^ins^ left diaphragm	0.20 ± 0.07 (0.18–0.23)	0.20 ± 0.06 (0.17–0.22)	0.945 (0.894–0.972)	0.015	0.042	0.224 *
T^exp^ left diaphragm	0.15 ± 0.05 (0.13–0.17)	0.13 ± 0.04 (0.12–0.15)	0.872 (0.672–0.942)	0.016	0.044	0.001 *
T^ins-exp^ left diaphragm	0.05 ± 0.04 (0.04–0.06)	0.06 ± 0.04 (0.05–0.08)	0.910 (0.791–0.957)	0.012	0.033	0.005 *
**Orthosis Device Probe Fixation**						
T^ins^ right diaphragm	0.23 ± 0.07 (0.20–0.25)	0.23 ± 0.08 (0.20–0.25)	0.982 (0.966–0.991)	0.001	0.002	0.468 *
T^exp^ right diaphragm	0.15 ± 0.05 (0.13–0.17)	0.16 ± 0.05 (0.12–0.15)	0.955 (0.907–0.977)	0.010	0.029	0.201 ^†^
T^ins-exp^ right diaphragm	0.07 ± 0.05 (0.05–0.09)	0.08 ± 0.05 (0.06–0.10)	0.936 (0.876–0.967)	0.012	0.035	0.211 ^†^
T^ins^ left diaphragm	0.22 ± 0.07 (0.19–0.24)	0.22 ± 0.07 (0.19–0.24)	0.979 (0.960–0.989)	0.010	0.027	0.840 *
T^exp^ left diaphragm	0.17 ± 0.05 (0.15–0.19)	0.15 ± 0.05 (0.12–0.16)	0.875 (0.523–0.952)	0.017	0.047	0.001 *
T^ins-exp^ left diaphragm	0.04 ± 0.05 (0.03–0.06)	0.05 ± 0.05 (0.03–0.07)	0.945 (0.893–0.972)	0.011	0.032	0.139 *

Abbreviations: CI, confidence interval; ICC, intraclass correlation coefficient; MDC, minimum detectable change; SD, standard deviation; SEM, standard error of measurement; T^ins^, maximum inspiration time; T^exp^, maximum expiration time. *p* < 0.05 was considered as statistically significant for a 95% CI (**in bold**). * Student *t*-test for paired samples was used. ^†^ Wilcoxon test for paired samples was used.

**Table 4:** Inter-rater and inter-session reliability analysis for RUSI diaphragm thickness within both manual and specific orthosis device measurement methods during relaxed breathing.

RUSI Diaphragm Thickness (cm)	Examiner 1 Mean ± SD (95% CI)	Examiner 2 Mean ± SD (95% CI)	ICC_(2,1)_ (95% CI)	SEM	MDC	*p*-Value
**Manual Probe Fixation**						
T^ins^ right diaphragm	0.21 ± 0.07 (0.19–0.24)	0.20 ± 0.07 (0.18–0.23)	0.965 (0.931–0.982)	0.013	0.036	**0.010 ^†^**
T^exp^ right diaphragm	0.13 ± 0.04 (0.11–0.15)	0.11 ± 0.04 (0.10–0.13)	0.865 (0.675–0.938)	0.014	0.040	0.002 *
T^ins-exp^ right diaphragm	0.08 ± 0.05 (0.06–0.10)	0.09 ± 0.06 (0.06–0.11)	0.953 (0.906–0.976)	0.014	0.039	0.047 *
T^ins^ left diaphragm	0.20 ± 0.07 (0.18–0.23)	0.20 ± 0.06 (0.17–0.22)	0.936 (0.876–0.967)	0.016	0.045	0.117 *
T^exp^ left diaphragm	0.15 ± 0.05 (0.13–0.17)	0.13 ± 0.05 (0.12–0.15)	0.784 (0.571–0.890)	0.023	0.064	0.025 *
T^ins-exp^ left diaphragm	0.05 ± 0.04 (0.04–0.06)	0.06 ± 0.03 (0.04–0.07)	0.828 (0.669–0.911)	0.014	0.040	0.172 *
**Orthosis Device Probe Fixation**						
T^ins^ right diaphragm	0.23 ± 0.07 (0.20–0.25)	0.22 ± 0.07 (0.19–0.24)	0.861 (0.731–0.928)	0.026	0.072	0.141 *
T^exp^ right diaphragm	0.15 ± 0.05 (0.13–0.17)	0.14 ± 0.05 (0.12–0.16)	0.927 (0.847–0.964)	0.013	0.037	0.410 ^†^
T^ins-exp^ right diaphragm	0.07 ± 0.05 (0.05–0.09)	0.07 ± 0.06 (0.05–0.09)	0.852 (0.713–0.924)	0.021	0.058	0.717 ^†^
T^ins^ left diaphragm	0.22 ± 0.07 (0.19–0.24)	0.20 ± 0.07 (0.18–0.23)	0.920 (0.839–0.959)	0.054	0.027	0.051 *
T^exp^ left diaphragm	0.17 ± 0.05 (0.15–0.19)	0.16 ± 0.05 (0.14–0.18)	0.877 (0.762–0.937)	0.017	0.048	0.106 *
T^ins-exp^ left diaphragm	0.04 ± 0.05 (0.03–0.06)	0.04 ± 0.05 (0.02–0.06)	0.884 (0.776–0.940)	0.017	0.047	0.687 *

Abbreviations: CI, confidence interval; ICC, intraclass correlation coefficient; MDC, minimum detectable change; SD, standard deviation; SEM, standard error of measurement; T^ins^, maximum inspiration time; T^exp^, maximum expiration time. *p* < 0.05 was considered as statistically significant for a 95% CI (**in bold**). * Student *t*-test for paired samples was used. ^†^ Wilcoxon test for paired samples was used.

## Data Availability

Raw data will be available upon corresponding author request.
